# Influence of Age, Circadian and Homeostatic Processes on Inhibitory Motor Control: A Go/Nogo Task Study

**DOI:** 10.1371/journal.pone.0039410

**Published:** 2012-06-25

**Authors:** Patricia Sagaspe, Jacques Taillard, Hélène Amiéva, Arnaud Beck, Olivier Rascol, Jean-François Dartigues, Aurore Capelli, Pierre Philip

**Affiliations:** 1 CNRS USR 3413 SANPSY (Sleep, Attention and NeuroPSYchiatrie), Université de Bordeaux, Bordeaux, France; 2 University Hospital Pellegrin, Bordeaux, France; 3 INSERM U897, ISPED, Bordeaux, France; 4 MEDES Institute for Space Medicine and Physiology, Toulouse, France; 5 Departments of Clinical Pharmacology and Neurosciences, Hospital and University Paul Sabatier of Toulouse, France INSERM Clinical Investigation Center INSERM 9302 and UMR 825, Toulouse, France; CSIC-Univ Miguel Hernandez, Spain

## Abstract

**Introduction:**

The contribution of circadian system and sleep pressure influences on executive performance as a function of age has never been studied. The aim of our study was to determine the age-related evolution of inhibitory motor control (i.e., ability to suppress a prepotent motor response) and sustained attention under controlled high or low sleep pressure conditions.

**Methods:**

14 healthy young males (mean age  = 23±2.7; 20–29 years) and 11 healthy older males (mean age  = 68±1.4; 66–70 years) were recruited. The volunteers were placed for 40 hours in “constant routine”. In the “Sleep Deprivation SD” condition, the volunteer was kept awake for 40 hours to obtain a high sleep pressure condition interacting with the circadian process. In the “NAP” condition, the volunteer adopted a short wake/sleep cycle (150/75 min) resulting in a low sleep pressure condition to counteract the homeostatic pressure and investigate the circadian process. Performances were evaluated by a simple reaction time task and a Go/Nogo task repeated every 3H45.

**Results:**

In the SD condition, inhibitory motor control (i.e., ability to inhibit an inappropriate response) was impaired by extended wakefulness equally in both age groups (P<.01). Sustained attention (i.e. ability to respond accurately to appropriate stimuli) on the executive task decreased under sleep deprivation in both groups, and even more in young participants (P<.05). In the NAP condition, age did not influence the time course of inhibitory motor control or sustained attention. In the SD and NAP conditions, older participants had a less fluctuating reaction time performance across time of day than young participants (P<.001).

**Conclusion:**

Aging could be a protective factor against the effects of extended wakefulness especially on sustained attention failures due to an attenuation of sleep pressure with duration of time awake.

## Introduction

The rhythms and demands of modern societies imply that many workers need to support optimal cognitive functioning throughout extended period including nighttimes while performing complex activities (e.g., health, security and transport). Moreover, extended work during the night is known to increase the risk of professional errors [1]. It is therefore important to study the impact of extended wakefulness on complex performance, i.e. executive functions.

Two major regulatory processes, the circadian system driven by the endogenous biological clock and the sleep-wake homeostatic process which is dependent on the duration of prior wakefulness (sleep pressure/sleep need), interact to regulate sleep and wakefulness according to nycthemeral variations. The circadian process regulates wake- and sleep-promoting mechanisms (timing, consolidation) [2].

Aging is associated with marked changes in the timing, consolidation and structure of sleep. Specifically, marked changes appear in sleep timing, quality and duration, such as decreases in sleep depth (measured by arousal threshold), sleep intensity (measured by slow wave activity (SWA)), sleep continuity (measured by awakenings during the night), and sleep duration [3]. This reduction in sleep need may reflect age-related changes in the homeostatic and/or circadian aspects of sleep regulation [4], [5]. From the circadian perspective, aging has been shown to be associated with a reduced circadian amplitude and a phase advance of the core body temperature rhythm and melatonin rhythm [5]. In parallel, from the sleep homeostatic perspective, aging has been associated with a reduction in daytime sleep propensity, maximal capacity for sleep [6], sleep continuity, and nocturnal slow wave sleep (SWS) [7]. Aging has also been shown to be associated with a less profound build-up of homeostatic sleep pressure as indexed by a reduced relative increase of frontal EEG delta activity in the elderly during recovery sleep [8], [9]. Moreover, older people display a shallower dissipation of sleep pressure, as indexed by reduced SWS and slow wave activity (SWA) dynamics across the night [10], [11].

Sleepiness and neurobehavioral functions have also been shown to depend on the interaction of homeostatic and circadian processes [12], [13], [14].

Many studies have shown that extended wakefulness impairs neurobehavioral performance (i.e., sustained attention) as assessed by a basic test of simple reaction time [15], [16]. An inter-individual vulnerability related to age has been described. Young people show a higher sensitivity to sleep loss than older people in terms of degradation of performances during the night [17], [18], [19], [20].

Two studies have shown that neither the homeostatic process [21], [22] nor the circadian process [22] can explain the nocturnal performance decrement during prolonged wakefulness. Studies designed to quantify circadian and homeostatic influences under controlled conditions on basic reaction time performance suggest that there are age-related changes in the circadian promotion of alertness, in the wake-dependent decline of alertness and/or in the interaction of both homeostatic and circadian processes [20]. Other studies [13], [23], [24] suggest that the attenuated impact of prior wakefulness in older people is more related to a relatively flattened circadian amplitude of time course of performance than to reduced homeostatic sleep pressure.

Inhibition of action is a major component of executive control (i.e., higher cognitive functions) to afford adapted behavioral responses [25]. Effectively, unexpected changes in the environment may require the suppression of prepotent or automatic actions that have become inappropriate. Behavioral inhibition is regularly required in any everyday action including in potentially life-threatening situations (e.g., to inhibit motor response to avoid an obstacle when driving). A dysfunction of inhibitory control has been reported in a variety of behavioral disorders sharing a common disinhibitory psychopathology such as obsessive-compulsive disorder [26], attention-deficit/hyperactivity disorder [27], schizophrenia [28], antisocial personality disorder, conduct disorder and substance use disorder [29], [30], [31]. Inhibitory control of behaviour has typically been localized to the right-lateralized prefrontal cortex (PFC), more particularly in the right inferior frontal gyrus region in neuroimaging studies [32], [33].

The experiments testing the effect of sleep deprivation on PFC-related executive functions show inconsistent results. Indeed, some studies report that sleep deprivation has adverse effects on decision making [34] and on neuropsychological tasks involving executive functions [35], [36], [37]. Conversely, others studies show no impact of sleep deprivation on executive functioning [38], [39], [40]. Therefore, the effects of sleep loss and time of day depend on the specific component of executive functioning tested, on the paradigm used [41]. The effect of sleepiness on motor inhibition has not been extensively studied. Nevertheless, individuals seem to experience difficulty in withholding an inappropriate response (i.e., inhibition failure) after total sleep deprivation [42], [43], when having poor sleep [44] or when suffering from an obstructive sleep apnea syndrome [45], [46]. To better understand the influence of sleep/wake regulation that contributes to human executive control is a key challenge for cognitive neurosciences.

To our knowledge, the contribution of circadian system and sleep pressure influences on motor inhibitory control as a function of age has never been studied. The aim of our study is to determine the age-related evolution of simple or executive performance under high or low sleep pressure conditions.

## Methods

### Participants

Twenty five healthy participants, 11 older [Age (±SD)  = 68±1.4 years, range 66–70 years] and 14 young participants [Age (±SD)  = 23±2.7 years, range 20–29 years], were recruited via advertisements (at Universities, organizations or hospitals of Bordeaux and Toulouse) or internet announcements.

Participants gave their written and informed consent to the study which was approved by the local ethics committee (committee for the protection of persons participating in biomedical research, Comité de Protection des Personnes (CPP) Sud-Ouest et Outre Mer III).

Exclusion criteria were medical, psychiatric, neurologic and sleep disorders as assessed by screening questionnaires. Volunteers with self-reported excessive daytime sleepiness (Epworth Sleepiness Scale, score ≥11) [47] or a sleep complaint such as sleep apnea or insomnia (Basic Nordic Sleep Questionnaire, items score <4) [48] as well as evidence of psychopathology on the Symptom Check List (SCL-90R score >59) were excluded from the study. Volunteers underwent a clinical interview with a sleep specialist and a nocturnal polygraphy to rule out any sleep disorders (e.g., sleep apnea) or organic disorders affecting sleep, poor sleep hygiene or abnormal usual sleep patterns. Other exclusion criteria were smoking, medication or drug consumption, night work or shift work, or transmeridian flight within 3 months prior to the study.

A neuropsychological assessment ensured that older volunteers had no motor-, attention- or memory-related impairments. A neuropsychologist assesses a set of informant-based items describing performance of activities of daily living [49], as it has been demonstrated that the history of decline in instrumental activities of daily living performance may precede the clinical diagnosis of dementia by more than 10 years [50]. Neuropsychological measures including global cognitive functioning (Mini-Mental State Examination) (MMSE) [51], memory test [52], verbal fluency (Isaacs Set Test), executive functions, cognitive flexibility and working memory (Trail Making Test (TMT)), and attention and executive functions (Wechsler Digit Symbol Substitution Test) were assessed.

Each participant was monitored for 7 days with actimeters (Actiwatch®, Cambridge Neurotechnology, United Kingdom) confirming normal sleep timing and sleep duration, and showing at least 85% mean sleep efficiency over a week to be recruited. Participants were instructed to maintain their usual-preferential sleep patterns (habitual sleep/wake timing and sleep duration) verified by actimetric recordings 3 days before each condition of the protocol.

They spent an adaptation night in the laboratory to familiarize them to sleep in a hospital environment with EEG recording.

### Study Design

**Figure 1:**
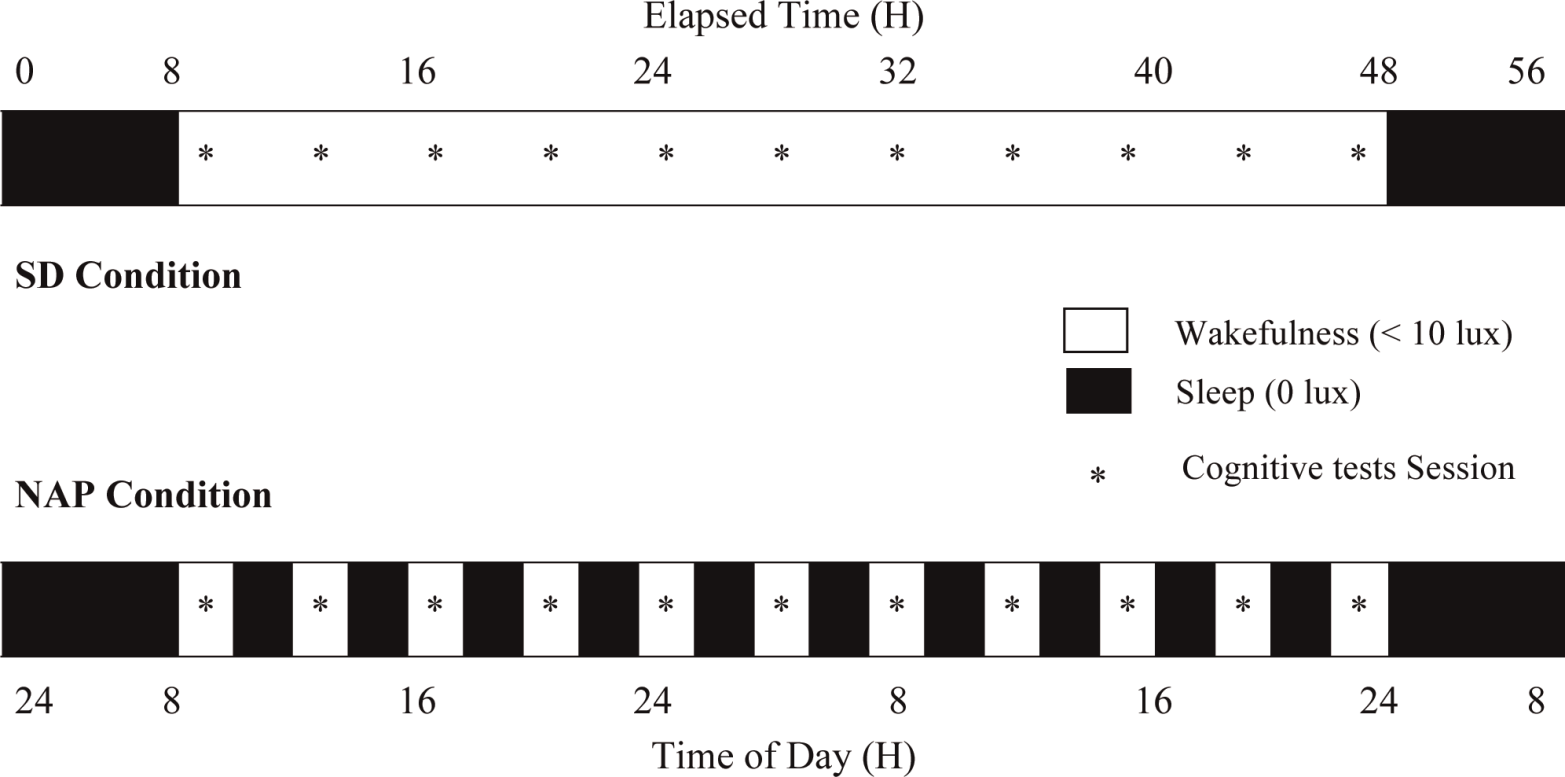
Overview of the protocol design [**13**]. After a baseline night, a 40-h Sleep Deprivation (SD) condition (top panel) and a 40-h NAP condition alternating short wake/sleep cycles (150/75 minutes) (lower panel) under constant routine protocol were carried out, followed by an 8-h recovery night.

Figure 1 represents the overview of the protocol design [13].

Each participant underwent 2 conditions, SD and NAP conditions (2 days each), in a balanced crossover design with a washout period of at least 2 weeks.

After a baseline sleep night, a 40-h SD under constant routine protocol or a 40-h NAP condition, both under constant conditions (semi-recumbent posture during scheduled wakefulness and supine during scheduled sleep/nap episodes, isocaloric snacks at regular intervals), was carried out [4], [13], [23], [37], [53], [54].

In the SD condition, the volunteers were kept awake during a 40-h extended wakefulness period to obtain a “high sleep pressure condition” interacting with the circadian process.

In the NAP condition, the volunteers adopted 10 alternating wake/sleep cycles (150/75 minutes) during a 40-h multiple sleep nap period resulting in a “low sleep pressure condition” to counteract homeostatic pressure and to examine the circadian influence.

A constant dim light level (<10 lux) during wakefulness and complete darkness (0 lux) during scheduled sleep/nap episodes were set. The protocol ended with an 8-h recovery sleep night.

Prior to the experiment, the participants were invited to complete training sessions to be familiarized with simple (Simple reaction time task SRTT) and executive tasks (Go/Nogo task) of the protocol.

The tests were performed 11 times every 3H45 throughout each condition.

### Simple Reaction Time Task

A 10-min simple reaction time test (SRTT) on a PALM personal organizer [55] was performed to evaluate sustained attention. A black square was displayed 100 times on the screen at randomized (2–7 s) intervals over 10 min. The subject was instructed to press a key as soon as the stimulus appears. This task was assessed every 3H45 (7H35, 11H20, 15H05, 18H50, 22H35, 2H20, 6H05, 9H50, 13H35, 17H20 and 21H05).

### Go/Nogo Task

The Go/Nogo task requires frequent automatic responding to stimuli interspersed with the need to suppress (i.e., to inhibit) a response from a specific, less frequently occurring stimulus.

The computerized Go/Nogo task is related to inhibitory functions and consists of 2 kinds of visual stimuli presented individually and in random order in the centre of the screen in white on a black background for 1250 ms preceded by a 250 ms fixation point and followed by a 500 ms interstimulus interval: 75% of Go stimuli (respond to a stimulus) and 25% of Nogo stimuli (refrain from responding to a stimulus). Thus a motor response had to be executed (Go) by pressing the space bar on the keyboard as quickly as possible, or inhibited (Nogo). The stimuli Go and Nogo (arrows to the left or to the right) were counter-balanced across participants. The experiment was programmed using E-Prime (v1.2, Psychology Software Tools, Inc., Pittsburgh, PA, USA, 2006). A total of 576 stimuli divided into 9 task blocks were shown during the 30 min task. This task was assessed every 3H45 (8H, 11H45, 15H50, 19H15, 23H, 2H45, 6H30, 10H15, 14H, 17H45 and 21H30).

### VAS Sleepiness

Immediately before each test sessions, participants were asked how sleepy they were on a 100-mm visual analogue scale (VAS), with scores ranging from 0 (“not sleepy at all”) to 100 (“very sleepy”).

### Data Analysis

Mean of the 10% slowest [56] (converted to reciprocal RTs) during the 10-min SRTT task were calculated. The outcome variables for the Go/Nogo performance included: Go RTs (response time for correct Go target); % missed Go (omission errors for Go target); % false positive Nogo (commission errors for Nogo stimuli). The ability to inhibit a prepotent motor response was measured with false positive rate (i.e., commission errors).

All variables were analyzed with three-way ANOVAs with repeated factors “condition” (SD vs. NAP), time (T 1–11) and the between subject-factor “age” (young vs. older). Planned comparisons were performed to localize statistical differences in significant main effect or interaction. Alpha criterion was set at P = .05. Statistica® (StatSoft Inc. 2010, Statistica for Windows, Maisons-Alfort, France, Version 9.1) was used.

## Results

### Sleep Parameters (Actimetric Recordings) before Conditions

No significant difference appears on total sleep time before SD condition and NAP condition (Mean ± SD  = 482±49 versus 474±56, respectively; Wilcoxon test, Z = 1.183, NS). No significant difference appears on sleep efficiency before SD condition and NAP condition (Mean ± SD  = 89±2.7 versus 89±3.3, respectively; Wilcoxon test, Z = 0.484, NS).

### Simple Task: Simple Reaction Time Task (SRTT)

#### 10% slowest RTs

**Figure 2:**
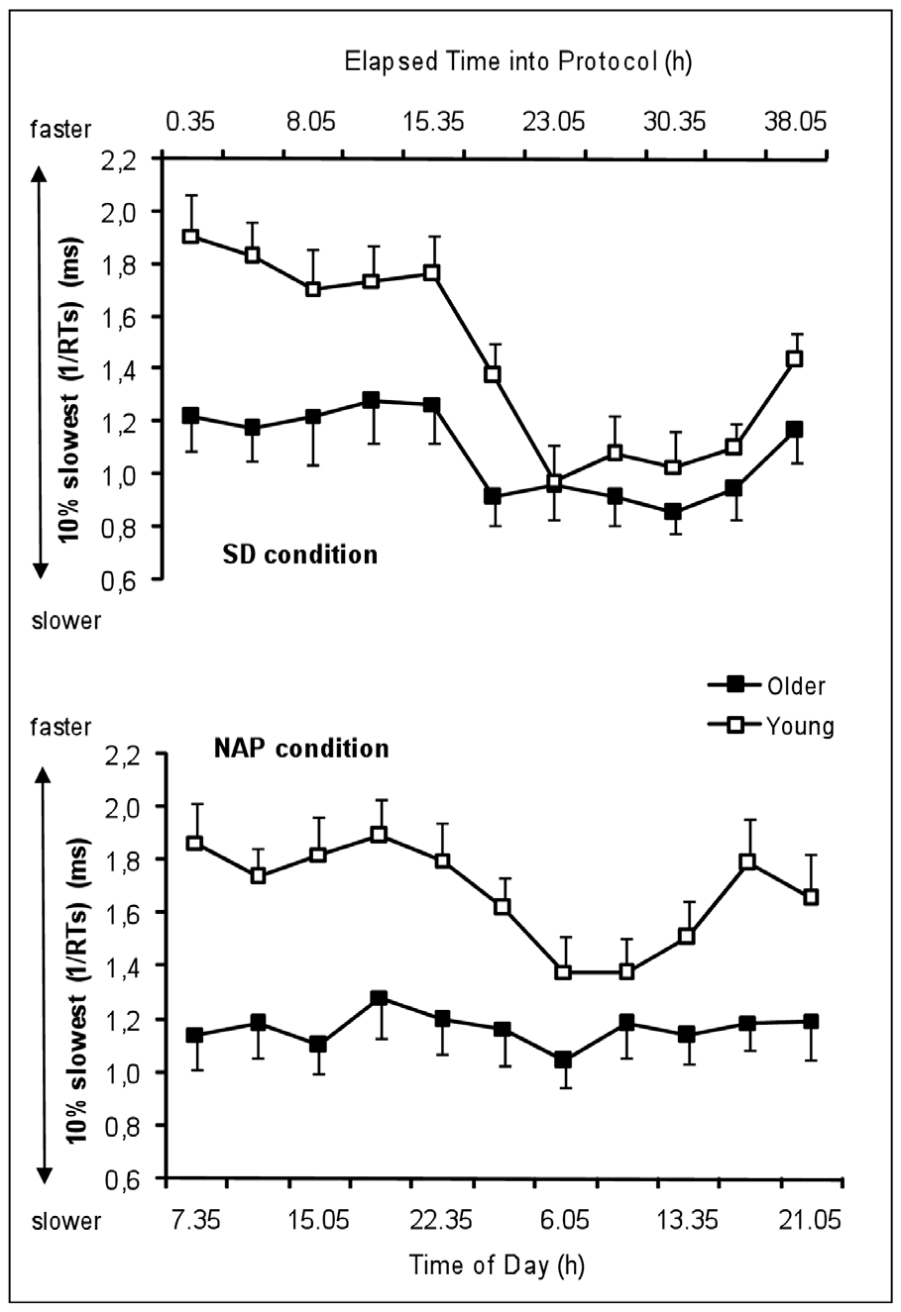
Time course of the 10% slowest reaction times (1/RTs) in the SRTT of the young and the older group under SD and NAP conditions (mean values ± SEM). SD  =  Sleep deprivation SRTT  =  Simple reaction time task.

Figure 2 represents the time course of the 10% slowest RTs of the young and the older group under SD and NAP conditions. Table 1 summarizes the results of the rANOVA (main effects and interactions) on 10% slowest reaction times.

**Table 1:** Results of the rANOVAs of the measures “VAS Sleepiness scores” (KSS), “10% slowest RTs” (SRTT), “Go RTs”, “% missed Go”, “% false positive Nogo” (Go/Nogo task).

		VAS		SRTT		Go/Nogo					
Effect	d.f.	Subjective Sleepiness		10% slowest RTs		Go RTs		% missed Go		% false positive Nogo	
		*F*-value	*P*-value	*F*-value	*P*-value	*F*-value	*P*-value	*F*-value	*P*-value	*F*-value	*P*-value
**Age**	1, 23	1.5	NS	9.3	**<.01**	5.3	**<.05**	4.0	= .056	3.6	= .070
**Condition**	1, 23	5.7	**<.05**	11.6	**<.01**	5.9	**<.05**	24.9	**<.001**	6.6	**<.05**
**Time**	10, 230	14.0	**<.001**	16.0	**<.001**	10.7	**<.001**	8.8	**<.001**	5.8	**<.001**
Age*Condition	1, 23	0.4	NS	2.5	NS	1.0	NS	3.3	= .082	0.4	NS
Age*Time	10, 230	2.6	**<.01**	4.2	**<.001**	4.7	**<.001**	2.2	**<.05**	1.3	NS
Condition*Time	10, 230	5.4	**<.001**	4.1	**<.001**	5.3	**<.001**	8.7	**<.001**	2.6	**<.01**
Age*Condition*Time	10, 230	2.6	**<.01**	0.7	NS	0.9	NS	1.9	**<.05**	1.2	NS

d.f.  =  Degree of Freedom.

VAS  =  Visual analog scale.

SRTT  =  Simple Reaction Time Task.

The main effect “age” yielded significance for the 10% slowest RTs (F_1,23_ = 9.3, P<.01) with significantly slower reaction times in older than young participants. The main factor “condition” was significant (F_1,23_ = 11.6, P<.01) with significantly slower reaction times in SD condition than NAP condition. The main factor “time” was significant (F_10,230_ = 16.0, P<.001) with significantly slower reaction times during (P<.001) and after (P<.001) the biological night compared to the baseline day. The factor “time” significantly interacts with the factor “condition” (F_10,230_ = 4.1, P<.001) with a slowing of reaction times after the biological night more pronounced in the SD than in the NAP condition. The factor “age” did not significantly interact with the factor “condition” (F_1,23_ = 2.5, NS), but with the factor “time”, with young participants becoming as slow as older participants at the end of the biological night and during the subsequent day in the SD and NAP conditions (F_10,230_ = 4.2, P<.001), except in the late afternoon (17H20: P<.05 and 21H05: P<.05). The interaction “age”, “condition”, “time” did not yield any significance.

### Executive Task: Go/Nogo task

#### Go RTs

**Figure 3:**
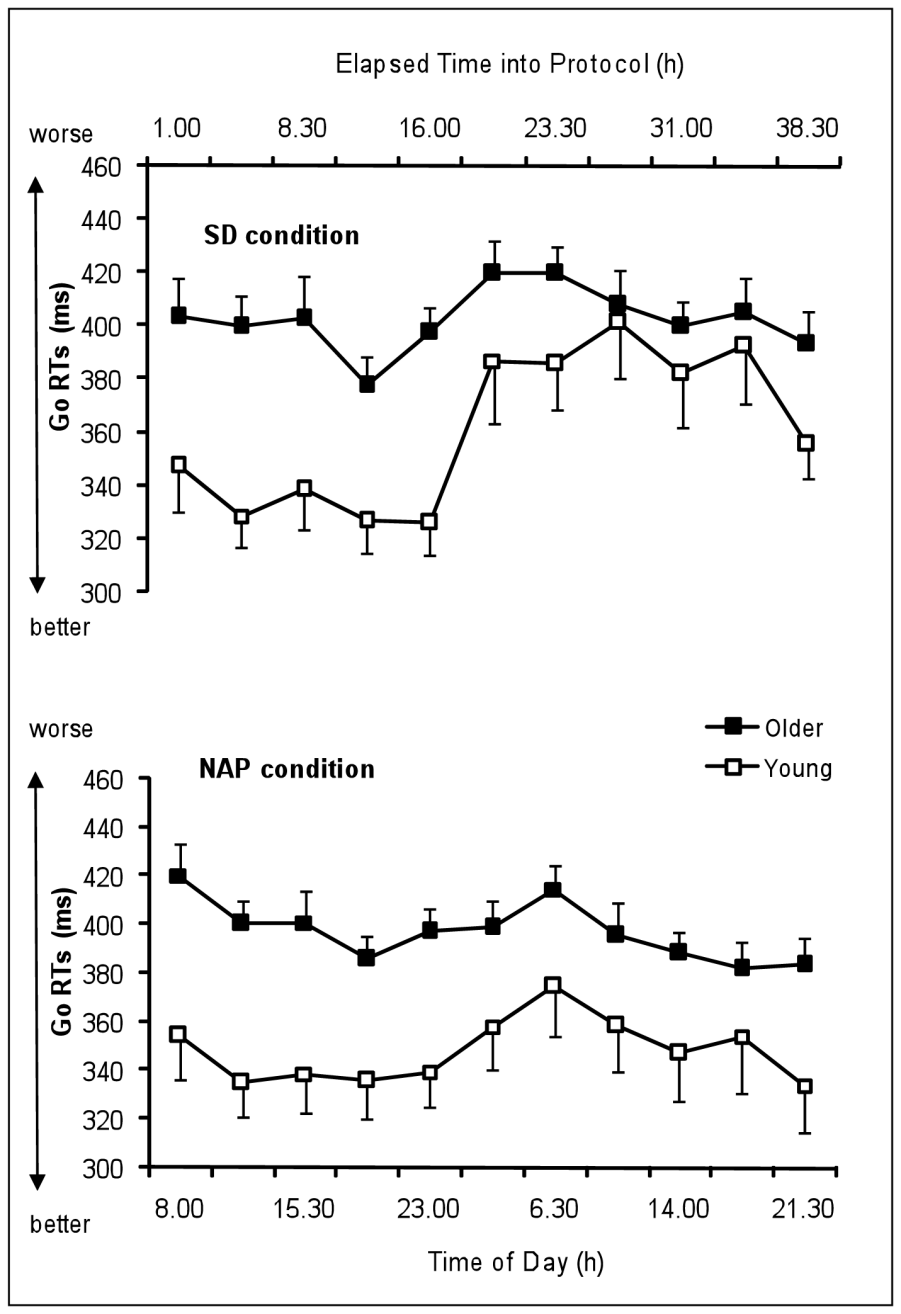
Time course of the Go RTs in the Go/Nogo task of the young and the older group under SD and NAP conditions (mean values ± SEM). SD  =  Sleep deprivation.

Figure 3 represents the time course of Go RTs of the young and the older group under SD and NAP conditions. Table 1 summarizes the results of the rANOVA (main effects and interactions) on Go RTs.

The main effect “age” yielded significance for Go RTs (F_1,23_ = 5.3, P<.05) with significantly slower reaction times in older than young participants. The main factor “condition” was significant (F_1,23_ = 5.9, P<.05) with significantly slower reaction times in SD condition than NAP condition. The main factor “time” was significant (F_10,230_ = 10.7, P<.001) with significantly slower reaction times during (P<.001) and after (P<.05) the biological night compared to the baseline day. The factor “time” significantly interacts with the factor “condition” (F_10,230_ = 5.3, P<.001) with a slowing of reaction times after the biological night more pronounced in the SD than in the NAP condition. The factor “age” did not significantly interact with the factor “condition” (F_1,23_ = 1.0, NS), but with the factor “time”, with young participants becoming as slow as older participants at the end of the biological night and during the subsequent day in the SD and NAP conditions (F_10,230_ = 4.6, P<.001), except in the evening (21H30: P<.05). The interaction “age”, “condition”, “time” did not yield any significance.

#### % missed Go

**Figure 4:**
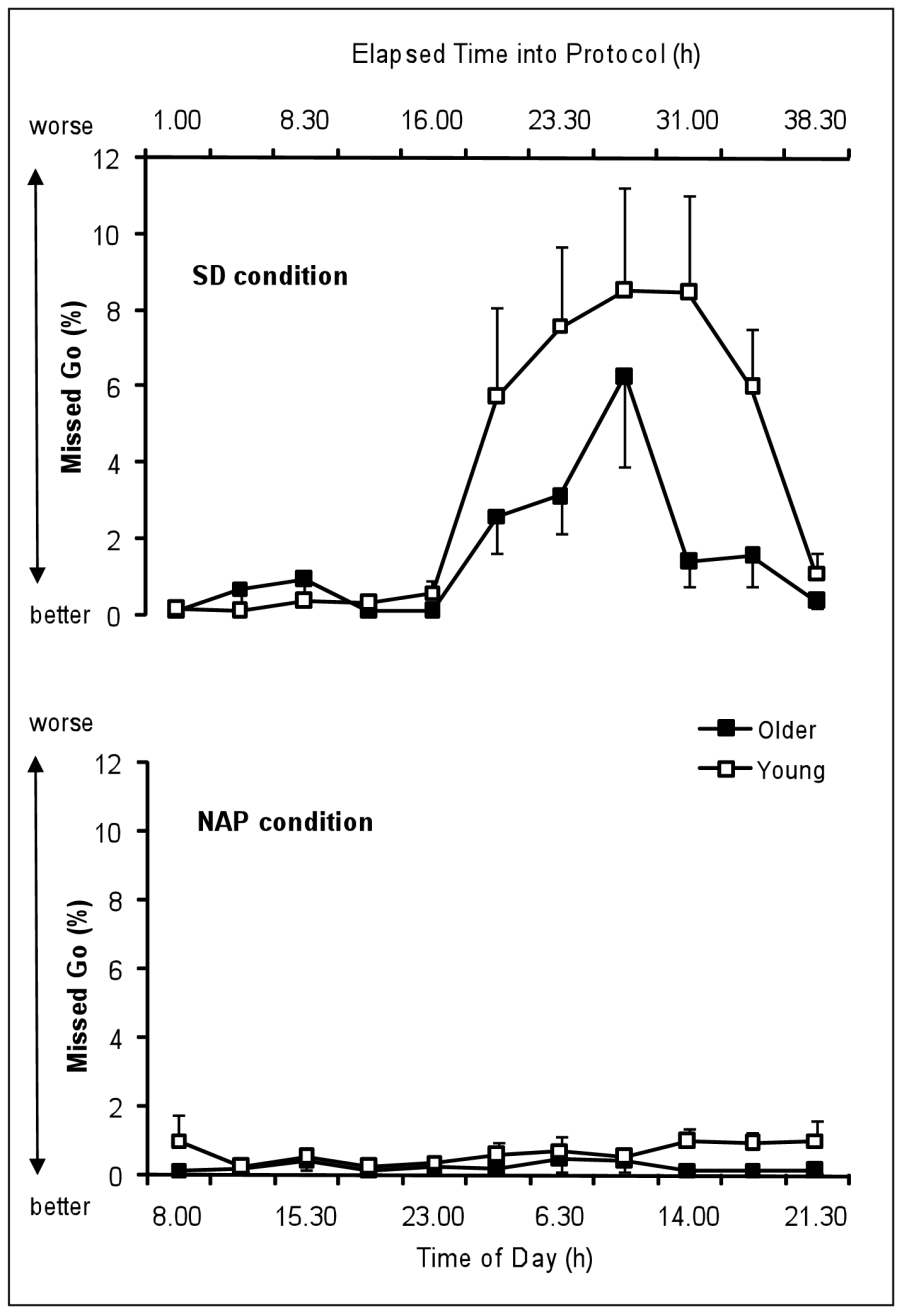
Time course of the percentage of missed Go in the Go/Nogo task of the young and the older group under SD and NAP conditions (mean values ± SEM). SD  =  Sleep deprivation.

Figure 4 represents the time course of percentage of missed Go of the young and the older group under SD and NAP conditions. Table 1 summarizes the results of the rANOVA (main effects and interactions) on % missed Go.

It is to note that two young participants out of the 25 participants did not miss any Go trial in the overall of the NAP condition.

The main effect “age” yielded significant tendency for the % missed Go (F_1,23_ = 4.0, P = .056) with higher % missed in young than older participants. The main factor “condition” was significant (F_1,23_ = 24.9, P<.001) with significantly higher % missed in SD condition than NAP condition. The main factor “time” was significant (F_10,230_ = 8.8, P<.001) with significantly higher % missed during (P<.01) and after (P<.001) the biological night compared to the baseline day. The factor “time” significantly interacts with the factor “condition” (F_10,230_ = 8.7, P<.001) with higher % missed during and after the biological night exclusively in the SD condition. The factor “age” did not significantly interact with the factor “condition” (F_1,23_ = 3.3, P = 0.08), but with the factor “time” (F_10,230_ = 2.2, P<0.05). The interaction “age”, “condition”, “time” yielded significance (F_10,230_ = 1.9, P<0.05). Planned comparisons show that young participants made higher % missed than older participants during the subsequent day after the biological night in the SD condition (14H: P<.05 and 17H45: P<.05) while age group difference was inexistent in the NAP condition.

#### % false positive Nogo

**Figure 5:**
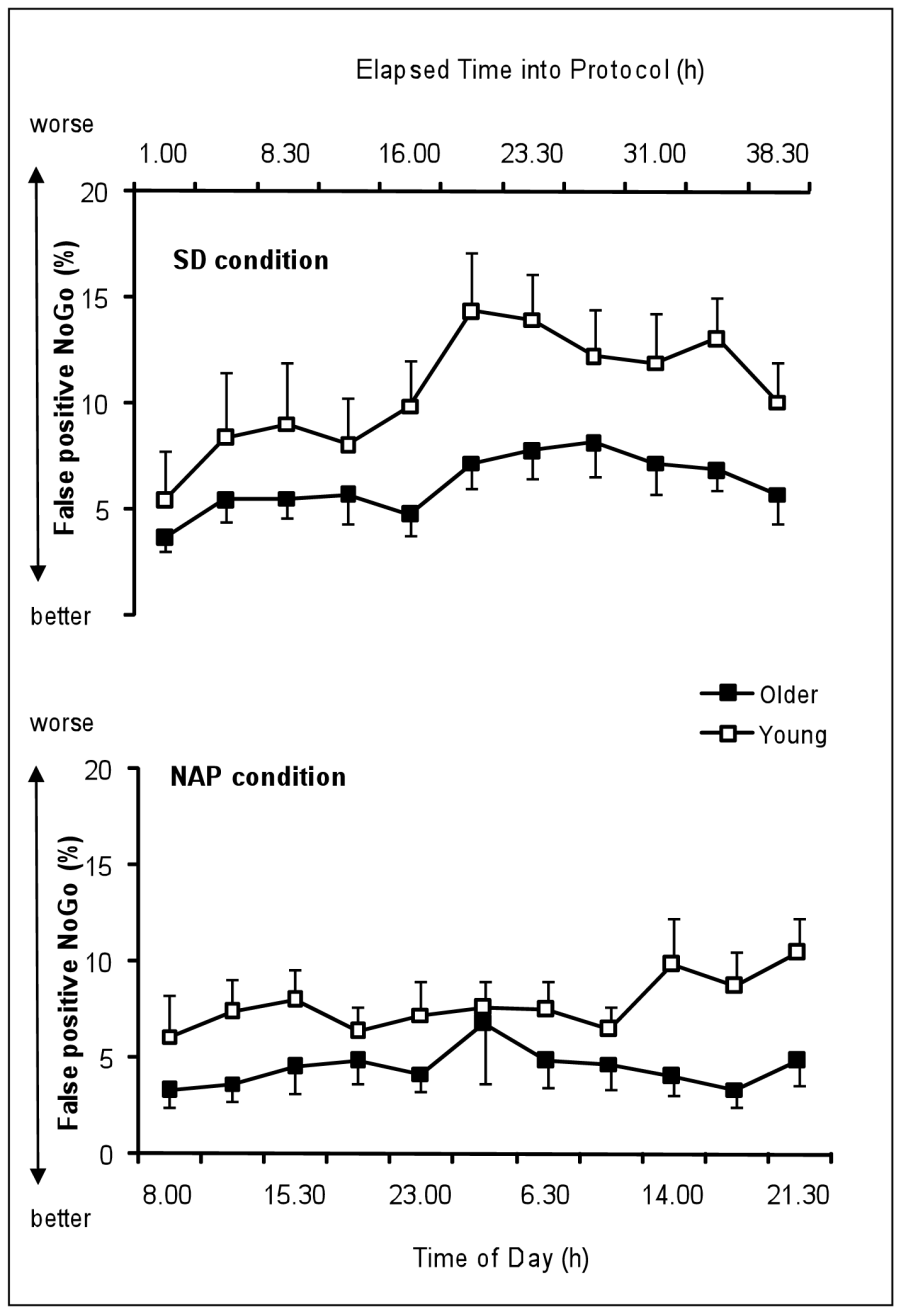
Time course of the percentage of % False Positive Nogo in the Go/Nogo task of the young and the older group under SD and NAP conditions (mean values ± SEM). SD  =  Sleep deprivation.

Figure 5 represents the time course of percentage of false positive Nogo of the young and the older group under SD and NAP conditions. Table 1 summarizes the results of the rANOVA (main effects and interactions) on % false positive Nogo.

The main effect “age” did not yield significance for the % false positive Nogo (F_1,23_ = 3.6, P = 0.07). The main factor “condition” was significant (F_1,23_ = 6.6, P<0.05) with significantly higher % false positive Nogo in SD condition than NAP condition. The main factor “time” was significant (F_10,230_ = 5.8, P<0.001) with significantly higher % false positive Nogo during (P = .057) and after (P<.01) the biological night compared to the baseline day. The factor “time” significantly interacts with the factor “condition” (F_10,230_ = 2.6, P<.01) with higher % false positive Nogo during and after the biological night in the SD than in the NAP condition. The factor “age” did not significantly interact with the factor “condition” (F_1,23_ = 0.4, NS) nor with the factor “time” (F_10,230_ = 1.3, NS). The interaction “age”, “condition”, “time” did not yield any significance (F_10,230_ = 1.2, NS).

### Subjective Sleepiness

#### VAS Sleepiness

Table 1 summarizes the results of the rANOVA (main effects and interactions) on VAS subjective sleepiness scores.

The main effect “age” did not yield significance for the VAS Sleepiness (F_1,23_ = 1.5, NS). The main factor “condition” was significant (F_1,23_ = 5.7, P<.05) with significantly higher subjective sleepiness scores in SD condition than NAP condition. The main factor “time” was significant (F_10,230_ = 14.0, P<.001) with significantly higher subjective sleepiness scores during (P<.001) and after (P<.001) the biological night compared to the baseline day. The factor “time” significantly interacts with the factor “condition” (F_10,230_ = 5.4, P<.001) with higher subjective sleepiness scores during (P<.05) and after (P<.001) the biological night compared to the baseline day, which were more pronounced in the SD than in the NAP condition. The factor “age” did not significantly interact with the factor “condition” (F_1,23_ = 0.4, NS), but with the factor “time” (F_10,230_ = 2.6, P<.01). The interaction “age”, “condition”, “time” yielded significance (F_10,230_ = 2.6, P<.01). Planned comparisons show that young participants estimate themselves less sleepy than older participants during the day following normal sleep while youngest become as sleepy as older participants during the biological night in the SD condition. No age group difference did exist in the NAP condition.

## Discussion

Our study confirms that normal aging leads to a cognitive slowing (i.e., increased reaction time) in simple and complex tasks [57]. However, we observe that accuracy in a behavioral inhibition task (i.e., Go/Nogo task) is fully preserved in older people [58]. Indeed, during the first day of the experiment after a normal sleep night, no difference appears on accuracy performance (i.e., errors of omissions (missed Go target) and commissions (false positive response to Nogo stimuli)) between young and older groups. As suggested by previous studies [59], older people do not present inhibitory motor control deficit in Go/Nogo task compared to young individuals.

Regarding the influence of sleep deprivation on speed-related processing, we found a slowing of reaction time performance on simple and executive tasks during and after the biological night in the SD condition in both age groups, which was even more pronounced for young participants. The latter tend to become as slow as older participants at the end of the biological night and during the morning hours of the subsequent day. This could mean that the circadian process has a greater adverse effect on younger people than on older ones. Blatter et al. (2006) [23] conclude that the 10% slowest RTs increase was significantly less pronounced in the older people than in the young during the biological night (24 h-8 h), so that both age groups exhibited similar performance decrements after the biological night. Thus, sleep pressure-related RT slowing in the young “make them old”, or the older people are less susceptible to circadian and wake-dependent PVT performance decrements.

In addition, we observe that the older people’s performance curve follows a flattened time course under low sleep pressure in the NAP condition compared to that of young participants. Inasmuch as the condition (high vs. low sleep pressure) does not influence this pattern (interaction age*time*condition not significant), our study confirms that age-related lower vulnerability to extended wakefulness seems predominantly due to an attenuated circadian regulation on reaction time performance in the older group [4], [23], [54] especially in the late biological night as previously described [20]. We cannot rule out that age-related reduced motor abilities prevent any kind of circadian modulation due to a floor effect (i.e., the level of performance cannot be lowered).

Regarding accuracy performance, actions errors during a Go/Nogo task can result either from sustained attention failure (i.e., omission errors) or from inhibition failure (i.e., commission errors). The percentages of omission and commission errors are stable across day and night when sleep pressure is low (i.e., in the multiple naps condition). Our study shows that, conversely to reaction time performance, the accuracy on executive task, which represents the success criterion of correctly achieving a task, is not modulated by the circadian component. We observe a deterioration of accuracy performance under high sleep pressure (i.e., sleep deprivation condition). Indeed, our results show that young and older individuals experience difficulty in ability to inhibit an inappropriate prepotent response (i.e., inhibition failure) and difficulty in responding accurately to appropriate stimuli (i.e., sustained attention failure) during and after a night of sleep deprivation. These results corroborate those of Drummond et al. (2006) [42] regarding the impaired ability in young people to inhibit a response in a Go/Nogo task after one night of total sleep deprivation. It is noteworthy that the difficulty in responding accurately to appropriate stimuli (i.e., sustained attention failure) under sleep deprivation is amplified in young compared to older participants. As suggested by other studies [8], homeostatic sleep pressure would be lower in the older people, allowing them to be less vulnerable to sustained attentional failure after a night of sleep deprivation. For the first time to our knowledge, as no circadian variation was observed in the multiple naps condition, our results show that an increase in errors in an executive task under extended wakefulness depends principally of the effect of increasing sleep pressure with duration of time awake. However, we can not totally exclude that an amplification of the circadian process occurs by increasing homeostatic sleep pressure. It is to note that nap should be effective countermeasures to sleepiness on the accuracy component of a task, particularly in young individuals [60].

Here, we used a constant routine protocol that constitutes the gold standard to measure circadian modulation of neurobehavioral functions, as well as the effect of sleep pressure developing with duration of time awake. In addition, the condition of scheduled sleep at regular intervals during a 40-h episode makes it possible to maintain low sleep pressure conditions and thus reveals the circadian rhythm without the confounding effects of elevated sleep pressure. However, further studies using a forced desynchrony protocol are needed to identify the contribution of the homeostatic and circadian processes on performance.

Moreover, we evaluate the effects of age, circadian and homeostatic influences on behavioral inhibition (i.e., ability to suppress a prepotent response) through commission errors on a Go/Nogo task. Further studies will have to evaluate others aspects of response withholding as the ability to stop a response that has already been initiated (e.g., Stop signal paradigm) [61].

Our study confirms the importance of circadian and homeostatic factors in the regulation of neurobehavioral function. However, in addition to the age factor, the characteristics of the tasks (simple or executive) [37] and the variables analyzed (speed or error-related component) is to be considered in light of the differential effects exerted by the circadian and homeostatic processes.

In conclusion, we show that inhibitory motor control (i.e., suppression of an inappropriate prepotent motor response) is fully preserved in no sleep-deprived aged people while equally impaired by extended wakefulness in young and older people. Our study reveals that error-related processing in a behavioral inhibition task does not seem to be regulated by circadian processes contrary to speed-related processing. Moreover, older people demonstrate not only an attenuation of the circadian influence on speed-related processing but also a reduction of sleep pressure with duration of time awake on sustained attention error-related processing. Therefore, aging could be a protective factor against the effects of extended wakefulness on sustained attention failures due to an attenuation of sleep pressure with duration of time awake. Strategies could be developed to prevent accidents according to the age of workers and their work schedule.
